# Recurrence-mediated suprathreshold stochastic resonance

**DOI:** 10.1007/s10827-021-00788-3

**Published:** 2021-05-18

**Authors:** Gregory Knoll, Benjamin Lindner

**Affiliations:** 1 Bernstein Center for Computational Neuroscience Berlin, Philippstr. 13, Haus 2, Berlin, 10115 Germany; 2 Physics Department of Humboldt University Berlin, Newtonstr. 15, 12489 Berlin, Germany

**Keywords:** Suprathreshold stochastic resonance, Recurrence, Spiking networks, Signal encoding

## Abstract

It has previously been shown that the encoding of time-dependent signals by feedforward networks (FFNs) of processing units exhibits suprathreshold stochastic resonance (SSR), which is an optimal signal transmission for a finite level of independent, individual stochasticity in the single units. In this study, a recurrent spiking network is simulated to demonstrate that SSR can be also caused by network noise in place of intrinsic noise. The level of autonomously generated fluctuations in the network can be controlled by the strength of synapses, and hence the coding fraction (our measure of information transmission) exhibits a maximum as a function of the synaptic coupling strength. The presence of a coding peak at an optimal coupling strength is robust over a wide range of individual, network, and signal parameters, although the optimal strength and peak magnitude depend on the parameter being varied. We also perform control experiments with an FFN illustrating that the optimized coding fraction is due to the change in noise level and not from other effects entailed when changing the coupling strength. These results also indicate that the non-white (temporally correlated) network noise in general provides an extra boost to encoding performance compared to the FFN driven by intrinsic white noise fluctuations.

## Introduction

The role of neurons as signal encoders has been shown to be enhanced in some situations by noise through the phenomenon known as stochastic resonance (SR), in which a finite amount of noise linearizes the response of a single processing unit or a population to a weak signal (Gammaitoni et al., 1998; Lindner et al., 2004; McDonnell & Ward, 2011). The necessary stochasticity in single neurons can arise from channel noise (Steinmetz et al., 2000; Schmid et al., 2004; Fisch et al., 2012), background synaptic input (Calvin & Stevens, 1968; Shadlen & Newsome, 1994; van Vreeswijk & Sompolinsky, 1996), or in networks of cells through heterogeneity (Chelaru & Dragoi, 2008; Marsat & Maler, 2010; Metzen & Chacron, 2015). Although the sources of noise abound, this type of noise-mediated coding benefit is largely limited to signals which are *subthreshold*, meaning that without noise they would not drive the cell to fire and would go undetected.

Stocks (2000) found that feedforward networks (FFNs) exhibit another distinct resonance from additive noise if it produces an effective heterogeneity in the response of the individual units, which aids in the encoding of both weak and strong signals and is therefore called *suprathreshold* stochastic resonance (SSR). Because neurons in the periphery have little if any recurrence and can be represented by FFNs, SSR has implications for biological encoding and has been demonstrated for a variety of models, including FitzHugh-Nagumo models (Stocks & Mannella, 2001; Hunsberger et al., 2014), nonlinear threshold devices (Hoch et al., 2003; Das et al., 2009), Hodgkin-Huxley models (Hoch et al., 2003; Ashida & Kubo, 2010; Hunsberger et al., 2014) and leaky integrate-and-fire (LIF) neurons (Hoch et al., 2003; Nikitin et al., 2010; Durrant et al., 2011; Hunsberger et al., 2014; Beiran et al., 2017). It has been shown to occur for different sources of variability, including channel fluctuations (Ashida & Kubo, 2010), intrinsic white current noise (Nikitin et al., 2010; Durrant et al., 2011; Hunsberger et al., 2014; Beiran et al., 2017), and heterogeneity (Stocks, 2000; Hunsberger et al., 2014; Beiran et al., 2017), as well as for signals with different distributions (Das et al., 2009) and correlations (Durrant et al., 2011).

In the cortex, the neurons in a population are no longer feedforward and receive inputs from many sources, including from other cortical regions (top-down), from FFNs in the sensory periphery (bottom-up), and from local, lateral, recurrent connections with one another. The top-down and bottom-up connections uncorrelated with a given signal can again be modeled as background synaptic input (conventional SR with respect to such external noise from other populations was for instance studied by Droste and Lindner (2017b)), but the recurrent inputs are no longer uncorrelated with one another or the signal. The resulting interactions are nonlinear (Abbott & van Vreeswijk, 1993; Brunel, 2000) and this *network noise* can become strongly colored, i.e. temporally correlated (Lerchner et al., 2006; Litwin-Kumar & Doiron, 2012; Ostojic, 2014) with correlation statistics that obey self-consistent relationships (Lerchner et al., 2006; Dummer et al., 2014).

Recurrent synaptic input is often modeled as a Gaussian white noise process through the *diffusion approximation* (Gluss, 1967; Johannesma, 1968; Capocelli & Ricciardi, 1971; Ricciardi, 1977; Brunel, 2000; Richardson, 2004). The current study therefore investigates signal encoding in recurrent networks and whether SSR can result from this synaptic network noise in place of intrinsic white noise. Once SSR is shown to occur, the intrinsic, network, and signal parameters will be varied in order to assess its robustness and the influence each type of parameter has on the magnitude and placement of its peak. Finally, we test whether the observed encoding could be obtained in control experiments in which the network input is replaced by a constant input, adjusted such that neurons have the same firing rate as neurons in the recurrent network (RN), or by a constant input and an individual Gaussian noise, adjusted according to the diffusion approximation. Only in the latter case does one observe an enhanced encoding. This demonstrates that the optimal coding fraction at a nonzero synaptic amplitude is indeed due to the network noise and not due to other effects that a variation of coupling strength might entail.

**Fig. 1:**
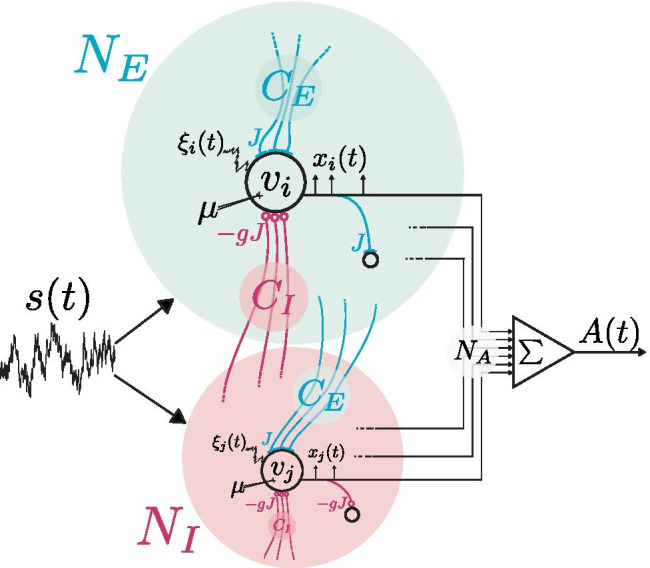
**System diagram. **The neural network is composed of $N_E$ excitatory cells (green) and $N_I$ inhibitory cells (red), all of which receive a common external stimulus *s*(*t*). An example of a single excitatory neuron *i* is shown above, with subthreshold voltage $v_i$ described by Eq. (1), intrinsic noise $\xi _i(t)$ and bias $\mu$. It also receives the common stimulus *s*(*t*) and local, lateral recurrent input randomly drawn from both populations: $C_E$ excitatory and $C_I$ inhibitory synapses. Excitatory neurons have a postsynaptic weight *J* and inhibitory neurons a postsynaptic weight of $-gJ$, regardless of the population to which the target belongs. The output of neuron *i* is spike train $x_i(t)$ (Eq. (2)), which elicits excitatory postsynaptic potentials with weight *J*, as shown for an example target neuron. An inhibitory cell *j* is also shown below, which only differs from *i* in its postsynaptic effect, meaning the input and firing statistics are equivalent for both excitatory and inhibitory neurons. As a result, $N_A$ arbitrarily chosen spike trains can be summed to calculate the population activity *A*(*t*) (Eq. (6))

## Network and neuron models

 The model (see Fig. 1) is a network of leaky integrate-and-fire neurons, whose individual subthreshold voltages $v_i$ evolve in time according to

1 $$\begin{aligned} \tau \dot{v}_{i} = -v_{i} + \mu + s(t) + \sqrt{2D_i \tau } \xi _i(t) + {RI_\text {i}(t)}, \end{aligned}$$

with a membrane time constant $\tau$, a mean constant input $\mu$, an intrinsic, Gaussian white noise with intensity $D_i \tau$ and correlation $\left\langle \xi _i(t)\xi _i(t') \right\rangle = \delta (t - t')$, a time-dependent stimulus *s*(*t*) common to all neurons, and recurrent input from the network ${RI_\text {i}(t)}$. For convenience, time can be measured in units of the membrane time constant and $\tau$ set to 1, with the noise scaled accordingly. When the voltage reaches the threshold value $v_T$=1 at time $t_k$, it is reset to the voltage $v_R$=0 and a spike is registered. The neuron then sits at the reset voltage for a refractory period $\tau _\text {ref}$. Throughout this paper, $\tau _\text {ref}$=0.1. The sum of all such spikes is the output, or *spike-train*, of the neuron

2 $$\begin{aligned} x_i(t) = \sum _k \delta (t - t_{i,k}), \end{aligned}$$

where $t_{i,k}$ denotes its $k^{\text {th}}$ spike time.

The stimulus *s*(*t*) is a Gaussian process with a flat power spectrum up to the cutoff frequency $f_c$

3 $$\begin{aligned} S_s\left( \ f \right) = \frac{\sigma _s^2}{2f_c} {\Theta }\left( \ f_c - \vert \ f \vert\right). \end{aligned}$$

Its variance is the integral over the power spectrum

4 $$\begin{aligned} \sigma _s^2 = \int _{-f_c}^{f_c} S\left( \ f \right) df. \end{aligned}$$

If $f_c$ is high enough, the signal is effectively Gaussian white noise and therefore the total white-noise intensity seen by the neuron (with $\tau =1$) is $\displaystyle D=D_i + \sigma _s^2/(4f_c)$.

Choosing instantaneous current-based synapses and a random network topology with fixed in-degree for simplicity, the input from the network is given by (see e.g. Brunel (2000) or Ostojic (2014))

5 $$\begin{aligned} {RI_\text {i}(t)} = \tau \sum _{j} J_{i j} \sum _{k} \delta \left( t-t_{j,k}-{\tau _{D, ij}} \right) , \end{aligned}$$

a sum over the spikes from neurons *j* providing input to the postsynaptic neuron *i*. The spikes arrive after a delay $\tau _{D, ij}$, which is independently drawn for each synaptic connection from a uniform distribution between 0.5ms and 2ms. Each of the *N* neurons in the network receives input from $C_E$ presynaptic neurons, whose spikes at time $t_{j,k}$ cause a positive, *excitatory* voltage excursion with weight $J_{ij} = J$, and from $C_I$ neurons whose spikes cause *inhibitory* voltage drops of magnitude $J_{ij}=-gJ$, i.e. *g* sets the relative strength of the spikes from inhibitory neurons. $C_E + C_I$ input neurons are selected randomly from the network for each neuron *i* such that there is no structure and because the number and the excitatory-to-inhibitory ratio are fixed, each neuron is subject to individual but statistically equivalent input. As there is no synaptic plasticity, this topology remains fixed throughout a single trial. Therefore, all neurons in the network have the same firing statistics, regardless of whether they are excitatory or inhibitory themselves. In addition, for each trial a new topology (realization of the network connectivity) is constructed and a new stimulus presented, such that averaging over trials constitutes averaging over intrinsic noise, random network connectivity, stimuli, synaptic delays and subpopulations (see below in Sect. (3)).

The network consists of $N = N_E + N_I$ neurons, $N_E$=10,000 excitatory and $N_I$=2,500 inhibitory, giving an inhibitory-to-excitatory ratio $\gamma =N_I/N_E=0.25$. The fraction of the population which provides input to the cells is given by $p_{c}$, such that $C_E=p_{c} N_E$ and $C_I=p_{c} N_I$ and therefore $C_I/C_E=\gamma$ as well.

## Measures

We are interested in the encoding fidelity of a group of neurons within the network, or how well a stimulus *s*(*t*) is received and transmitted in the activity

6 $$\begin{aligned} A(t) = \frac{1}{N_A} \sum _{i=1}^{N_A} x_i(t) \end{aligned}$$

of a population of neurons of size $N_A$. Because the output of all neurons is equivalent, the selection of the $N_A$ neurons in the population is arbitrary. The coherence function,

7 $$\begin{aligned} C_{As}\left( \ f \right) = \frac{\vert S_{As}\left( \ f \right)\vert^2}{S_A\left( \ f \right) S_s\left( \ f \right)}, \end{aligned}$$

quantifies the linear correlation between the signal and the activity in the Fourier domain. $S_{As}$ is the cross-spectrum between the activity and the stimulus

8 $$\begin{aligned} S_{As}\left( \ f \right) = \lim _{T \rightarrow \infty } \frac{\left\langle \tilde{A}\left( \ f \right)\tilde{s}^*\left( \ f \right) \right\rangle }{T} \end{aligned}$$

and $S_A\left( \ f \right)$ and $S_s\left( \ f \right)$ are the power spectra of the activity and stimulus, respectively, defined as

9 $$\begin{aligned} S_Y\left( \ f \right) = \lim _{T \rightarrow \infty } \frac{\left\langle \tilde{Y}\left( \ f \right)\tilde{Y}^*\left( \ f \right) \right\rangle }{T}. \end{aligned}$$

In the preceding equations, $Y^*$ is the complex conjugate, $\tilde{Y}$ denotes the Fourier transform $\int _0^T e^{i 2 \pi f t} Y(t) dt$, and $\left\langle \cdot \right\rangle$=$\langle \langle {\langle } \langle \langle \cdot \rangle _{\xi _i} \rangle _{J_{ij}} {\rangle _{\tau _{D, ij}}} \rangle _{s} \rangle _{N_A}$ is the average over intrinsic noise, network realizations (connectivity and synaptic delays), stimuli, and subpopulations.

In order to get a point estimate of the encoding fidelity over the entire frequency spectrum, the *coding fraction*

10 $$\begin{aligned} \Gamma = 1 - \sqrt{\frac{\displaystyle \int _{-\infty }^{\infty } S_s\left( \ f \right) [1 - C_{As}\left( \ f \right)] df}{\displaystyle \int _{-\infty }^{\infty } S_s\left( \ f \right) df}} = {1 - \frac{\epsilon }{\sigma _s}} \end{aligned}$$

is introduced, which measures the quality of the estimate of the stimulus from the activity (Gabbiani, 1996). The numerator in the square root is the mean square error $\epsilon ^2$ between the stimulus *s*(*t*) and its optimal linear reconstruction $s_\text {est}(t)$. The denominator is the variance of the signal, $\sigma _s^2$, making the entire square root expression the ratio of the standard deviations of the error and signal, $\epsilon /\sigma _s$, which will approach 0 for perfect reconstructions and approach 1 for estimates which are no better than chance. Therefore, like the coherence function, the coding fraction in Eq. (10) is limited to the range between 0 (chance resemblance) and 1 (the stimulus can be reconstructed in its entirety).

**Fig. 2:**
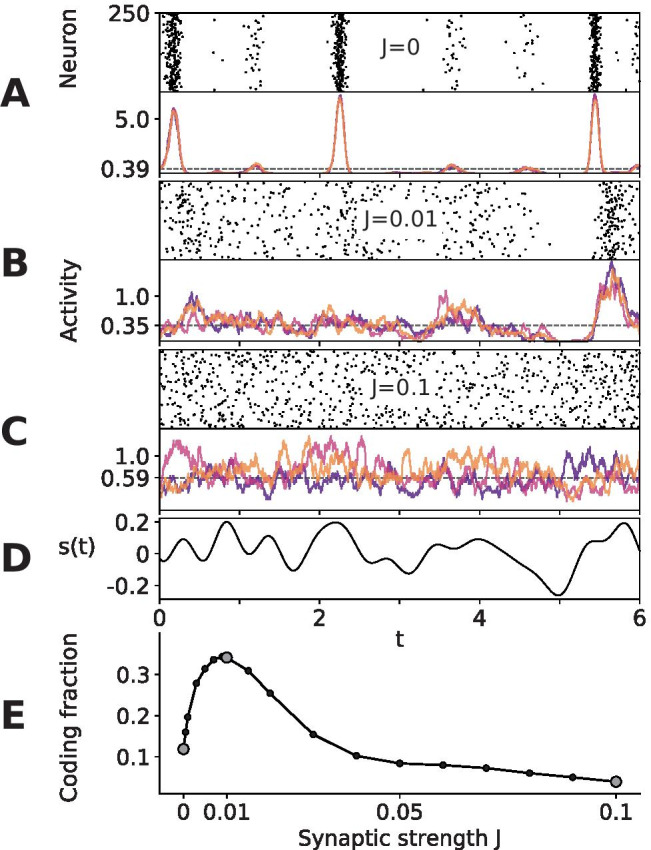
**Suprathreshold stochastic resonance resulting from an optimal amount of network noise.** **A-C: ** The raster plot of a subpopulation of $N_A$=250 neurons is shown above its activity from three trials, in which it was presented the same stimulus, *s*(*t*) (lowest panel). The dashed lines are the average firing rates. **D:** The stimulus *s*(*t*) was the same for the three synaptic strengths and their three trials. **E: ** The coding fraction versus synaptic strength, *J*. The filled markers correspond to the values shown in the time plots above. **Parameters:** $D_i$=$2.5\times 10^{-5}$, $\sigma _s$=0.1, $f_c$=2, $\mu$=1.1, *g*=5, $p_c$=0.01. T=200, dt=0.001, activity bin size $\Delta$=0.1. The coding fraction was calculated from 200 trials

## Evidence for suprathreshold stochastic resonance due to network noise

The network responses to a time-dependent stimulus for three different synaptic weights are illustrated in Fig. 2a-c, with the raster plot from a population of size $N_A$=250 plotted above its activity recorded at the same point in time from three trials. The same stimulus *s*(*t*) (Fig. 2d) was presented in all trials, not only to compare how the overall response changes with increasing weights, but also to demonstrate the trial-to-trial variability due to intrinsic and network noises.

Without recurrence (*J*=0; Fig. 2a), the only noise in the system is the very low intrinsic noise ($D_i$=$2.5\times 10^{-5}$) and the stimulus itself. The activity is therefore rather synchronous and shows rectification for negative signal swings. As the strength is increased to *J*=0.01 (Fig. 2b), the activity is more asynchronous and irregular, indicative of the network noise emerging due to the nonlinear interactions among the neurons. This noise is only weakly correlated for different neurons and thus endows each neuron with an independent individual response to the common driving stimulus. As a consequence, the entire population better captures the full swing of the stimulus and is able to encode more of the signal. In addition, the network noise remains low enough such that the population rate follows the signal reliably. At a much higher synaptic strength (*J*=0.1; Fig. 2c), there is even less rectification, but the network noise overpowers the signal, preventing reliable encoding. Hence, the intermediate value of the coupling strength in Fig. 2b seems to be optimal with respect to signal encoding. Because we mainly change the network noise when changing the synaptic coupling strength, this can be taken as evidence for the presence of a suprathreshold stochastic resonance effect. Of course, increasing the synaptic strength also changes the mean input to every neuron (and by that the firing rate) and there is also a network input related to the common stimulus. As we show in Sect. (5), however, taking these other connection-induced effects into account cannot explain the significant boost in the coding fraction.

The above conclusion is more systematically investigated in Fig. 2e, where the coding fraction is plotted as a function of synaptic strength. A new stimulus was presented in each trial, as indicated by the averages in Eq. (8) and Eq. (9) (the fixed stimulus in Fig. 2a-d was solely for illustration purposes). The coding fraction values corresponding to the time series in Fig. 2a-c are indicated by filled circles in the bottom panel. As stated above, with no synaptic input, there is little variability and therefore a low coding fraction. The coding fraction also suffers from large synaptic strengths, because the network noise drowns out the signal. The peak at *J*=0.01 represents suprathreshold stochastic resonance (SSR), where recurrence adds enough noise to improve encoding without dominating the neuronal dynamics.

**Fig. 3:**
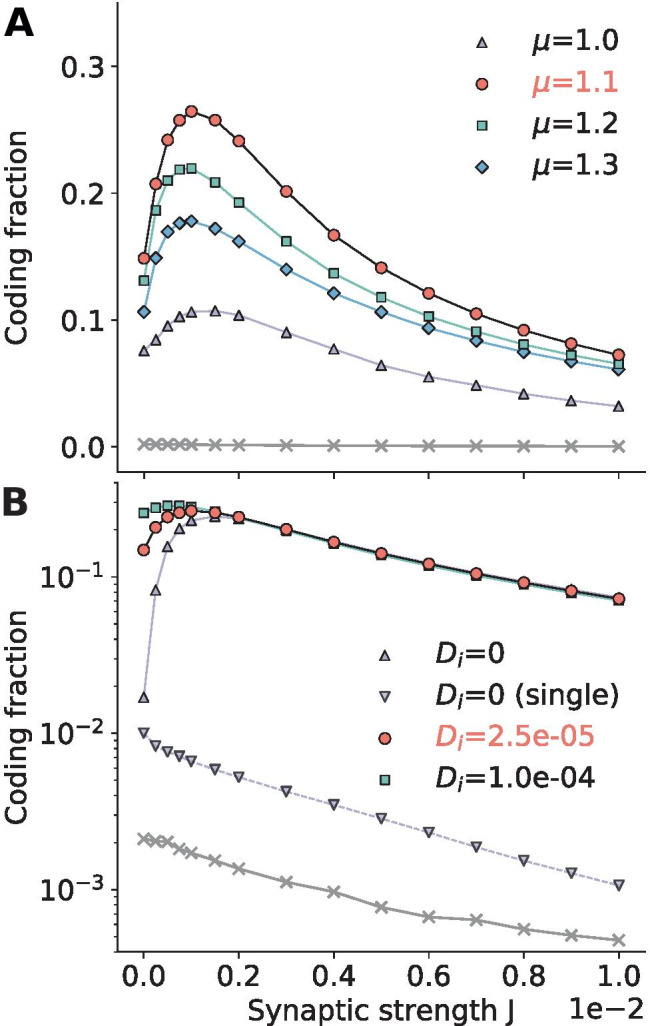
**Intrinsic parameters.** **A: ** The bias current is swept from $\mu$=1.0 (the threshold value) up to $\mu$=1.3, which is well into the suprathreshold range, revealing an optimal bias which maximizes the overall coding fraction. **B: ** The coding fraction without intrinsic noise ($D_i$=0; upturned triangles) is compared to two nonzero noise intensities. The single neuron’s coding fraction for the $D_i$=0 case is indicated by inverted triangles. **Both:** The red circles and labels are the default parameters and are the same in both panels. The x’s were calculated from the default parameter output and a random signal, indicating chance correlations. **Parameters (unless varied):** $\mu$=1.1, $D_i$=$2.5\times 10^{-5}$, *g*=5, $p_c$=0.01, $N_A$=250, $\sigma _s$=0.1, $f_c$=15

In the following we explore how the beneficial effect of the asynchronous state and the network noise found in Fig. 2 depends on the cellular, network, and signal parameters. Specifically, we will show how the dependence of the coding fraction on the coupling strength varies if we change these parameters.

### Intrinsic parameters

Although the neurons are embedded in a network, their individual properties will determine their responses to the signal and network activity. Two such properties are their individual bias current $\mu$ and their individual noise intensity $D_i$, both determining how often and how irregularly the single cell would fire without any network input and common stimulus. To one extreme, if the neurons are subthreshold ($\mu <v_T$) and have little intrinsic noise, they will barely be spontaneously active and will be slower to react to a signal, causing rectification similar to that seen in Fig. 2a in the absence of connectivity. As the neurons become more active, their output will increasingly amplify the network noise, which can also disrupt transmission if too strong.

That is why, as the bias $\mu$ is increased from just at threshold to well into the suprathreshold range, there is an optimal value which strikes the balance between actively sampling the signal and not driving the network too strongly. This is reflected in the global maximum of the family of curves for the coding fraction shown in Fig. 3a.

**Fig. 4:**
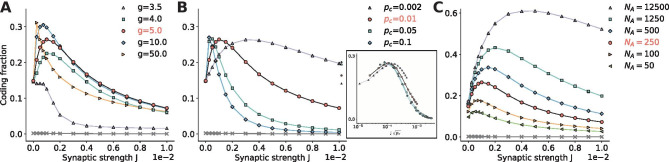
**Network parameters.** **A: ** The relative strength of the inhibitory synapses *g* shifts the excitatory-inhibitory mix in the synaptic input. The network is balanced if *g*=4, excitation-dominated for $g<$4, and inhibition-dominated for $g>$4. **B:** The connection probability $p_c$ represents the number of inputs to a single neuron, because the size of the network doesn’t change. It influences the amount of input as well as correlation entering a cell. The inset shows the same curves, but as a function of $J \sqrt{p_c}$ instead of *J*, demonstrating that the shifted peaks in the left panel represent the same amount of input, and are therefore equivalent. **C:** The size $N_A$ of the observed population is increased from $N_A$=50 up to the size of the entire network ($N_A$=*N*=12,500), each time improving the overall encoding. **Parameters (unless varied):** $\mu$=1.1, $D_i$=$2.5\times 10^{-5}$, *g*=5, $p_c$=0.01, $N_A$=250, $\sigma _s$=0.1, $f_c$=15

Increasing the intrinsic noise intensity improves the encoding at low synaptic strengths, as shown in Fig. 3b and found by Beiran et al. (2017) in the case of a pure feedforward population. The network of course benefits most from network noise if there is no intrinsic noise at all ($D_i$=0), because some noise is better than none. However, this only occurs for very weak coupling because as the synaptic strength increases (above J=0.002 in Fig. 3), the network noise dominates, diminishing or eliminating the benefits of relatively small amounts of added white noise at a given connection strength *J*.

Also included in Fig. 3b is the single neuron’s coding fraction for the $D_i$=0 case, indicated by inverted triangles; these values are all low. For $D_i$=0 and *J*=0, the signal is the only source of noise in the network, and it is common to all neurons. As a result, all neurons in the network are essentially identical and there is no benefit to taking a population’s instead of a single neuron’s output. The slight discrepancy in the coding fractions of the single neuron and population for *J*=0, $D_i$=0 is most likely due to the different initial conditions of the neurons in the population that lead to small differences in their spike trains at the point where measurements begin. We have verified that this artificial discrepancy can be reduced by increasing the initial transient period prior to measuring spikes for the coding fraction.

Because the mean input $\mu$=1.1 is above the threshold $v_T$=1, we do not observe common stochastic resonance in the single neuron’s coding fraction curve as the network noise is increased, lending evidence to the hypothesis that the observed transmission benefits seen in the other curves are due to suprathreshold stochastic resonance. As can be seen by comparing the two $D_i$=0 curves, a single neuron’s coding fraction only suffers further from network noise (monotonically decreases), whereas reading the population activity is immediately beneficial as soon as network noise is added, which is again an indication of suprathreshold stochastic resonance.

In order to aid comparison, the red circles with the black curves are the same in both panels of Fig. 3 and will be used throughout the paper as default parameters, against which other results are compared. The default value for the parameter of interest will be highlighted in red in the legend, as it is here. Parameters not specifically varied in a plot assume their default values. For instance, in Fig. 3a, $D_i$=2.5e-05, which is indicated in red in Fig. 3b, where D is varied.

The simulation data points marked by x’s in both panels are the result of calculating the coherence of the output of the network with default parameters and a random signal. The curve serves to better compare the measured coding fraction with chance values, serving as a type of lower bound on the coding fraction. Like the default parameter curve, it too will be shown in the following figures to add consistency and to serve as a reference to better assess the effects of varying a single parameter.

### Network parameters

We have seen that individual parameters can have an effect on the network performance. It is then of course interesting to see how the connectivity and size of the population amplify or detract from the collective performance.

As a point of reference, the relative inhibitory strength is set to *g*=5 in the default parameters, which causes slight inhibition-domination in order to put the network in the asynchronous irregular regime. It is intuitive that if $g<$4, the network will transition into the excitation-dominated regime and begin to oscillate, causing single neurons to synchronize (Brunel, 2000). Such pathological behavior will greatly diminish the network’s ability to listen to the signal and encode its information, which is reflected by the poor performance of the *g*=3.5 curve in Fig. 4a.

As the excitation-inhibition relation is shifted from balanced (*g*=4) to inhibition-dominated, the network noise increases in intensity and carries with it an increasingly negative mean recurrence. It is then a mixture of the effects from Fig. 3, where decreasing $\mu$ potentially improves the overall coding fraction, which can be seen most clearly by comparing *g*=4 and *g*=5 ($\mu$=1.1 here, so *g*=4 would be like the $\mu$=1.2 curve in Fig. 3a), and increasing the noise intensity improves the performance almost exclusively at lower synaptic strengths, as seen in the transition from *g*=5 to (the physiologically unrealistic value of) *g*=50.

Somewhat more surprising is that SSR is observed even at extreme inhibition levels, albeit for very low synaptic strengths. At those levels, the slightest synaptic strength gives the expected noise benefit because the network noise is still small, but further increases overwhelm the network and quickly degrade the encoding. If *J*=0, *g* of course has no effect and it can be seen in Fig. 4a that all curves are equivalent at this point.

Because the input from the network acts largely as noise and the amount of noise can be beneficial, it is reasonable to investigate whether changing the number of inputs instead of their strength has a significant impact on encoding. Fig. 4b shows that increasing the number of inputs shifts the peak to lower synaptic strengths and vice versa. However, the magnitude of the peak does not change significantly. In order to demonstrate this more clearly, the inset shows the coding fraction as a function of $J\sqrt{p_c}$ instead of *J*. The curves do show slight variations, but mostly for $p_c=0.002$ where there are so few inputs (only 25). Fig. 4b demonstrates that SSR is a result of the amount of network noise, and that the number of inputs that transmit that noise only serves to broaden the range of weights over which the peak extends.

In contrast, the number of neurons in the measured population does have a large effect on the magnitude and range of the coding fraction. SSR is the phenomenon of sampling diversity by a population of neurons of a common signal leading to a collective encoding advantage. In Fig. 4c this is demonstrated by increasing the size of the population from a relatively small number ($N_A$=50) to the entire network ($N_A$=12,500). All else being equal, the added degrees of freedom provided by larger sampling populations improve the encoding and the SSR peak, even up to very large population sizes.

**Fig. 5:**
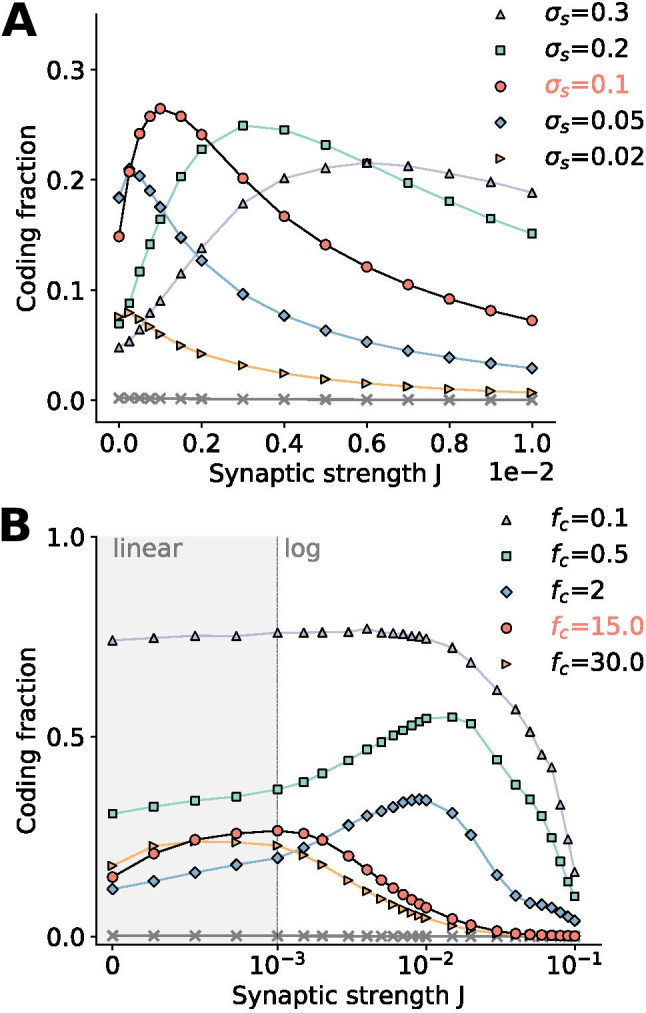
**Signal properties**. **A:** The standard deviation of the stimulus amplitude, $\sigma _s$, determines the relative strength of the signal compared to the system noise. **B: ** As the cutoff frequency is decreased, the stimulus information is concentrated with higher power in lower frequency bands and can be sampled more thoroughly. In order to show the peaks for lower cutoff frequencies, which shift the coding fraction peak to much higher synaptic weights, the data is plotted on a log scale above $10^{-3}$, while for smaller weights (gray region) the linear scale is preserved in order to include *J*=0. **Parameters (unless varied):** $\mu$=1.1, $D_i$=$2.5\times 10^{-5}$, *g*=5, $p_c$=0.01, $N_A$=250, $\sigma _s$=0.1, $f_c$=15

### Signal properties

So far the network and its units have been the focus, but the properties of the stimulus itself influence how well a subpopulation of the network can encode it. Signal parameters of interest are the amplitude (quantified by the standard deviation $\sigma _s$) and the bandwidth of the signal (quantified by the cutoff frequency $f_c$). We will inspect how changing those parameters with respect to our default values will affect the coding fraction.

First of all, whether the amplitude is large or small relative to the dynamics and noise of the network will determine the nonlinearity of the signal transfer and thus the value of the coding fraction. If, on the one hand, the signal is extremely weak, it will inadequately stimulate the system, as seen for $\sigma _s$=0.02 (orange right-pointing triangles in Fig. 5a), where the overall coding fraction is low. The slightest network noise drowns out the weak signal, shifting the small SSR peak to minuscule synaptic weights. On the other hand, if the amplitude is extremely large, the network response becomes strongly nonlinear because the stimulus drives all the neurons into synchronous firing or silences all of them at once. This kind of response is only weakly influenced by mutual synaptic connections within the network — silenced neurons, for instance, will not affect other cells, no matter how strong their outgoing synaptic connections are. The nonlinear all-or-none response is expected to lead to small values of the coding fraction, which, as we recall here, measures the *linear* part of the signal transfer. As a consequence, an intermediate amplitude might be optimal for the signal transmission.

These expectations are confirmed in Fig. 5a. As the stimulus amplitude is increased from very low values, the stimulus is well-encoded across a broad coupling range and the maximum is attained at a larger synaptic strength because (as known from conventional SSR) more noise is required for a stronger signal to maximize the coding fraction (Beiran et al., 2017). As was argued above, there is an optimal amplitude for a global maximum of the coding fraction, $\sigma _s$=0.1, shown by the curve with red points in Fig. 5a. For higher values of the stimulus amplitude, the SSR peak becomes broader and its maximum decreases (e.g. for $\sigma _s$=0.3; purple upturned triangles).

In addition to the stimulus amplitude, its frequency content must play a role, because the network has its own time scales governing how quickly it can react and recover. We recall that in our parametrization of the stimulus, if the variance $\sigma _s^2$ is fixed, then according to Eq. (4) an increase of the cutoff frequency $f_c$ implies that the spectral height $S_s\left( \ f \right)$ for $\vert \ f \vert<f_c$ will decrease, i.e. a lower density in a broader bandwidth. Therefore, as the cutoff frequency is increased from $f_c$=15 to $f_c$=30, it behaves like a weak signal within the frequency range for which the neuron is sensitive (compare orange right-pointing triangles in Fig. 5a and Fig. 5b). A decrease in the encoding of relatively high-frequency signals is also in keeping with the known low-pass-filter nature of LIF neurons and other integrators (Fourcaud-Trocmé et al., 2003; Vilela & Lindner, 2009).

At the other extreme, for $f_c$=0.1 (purple upturned triangles in Fig. 5b) the overall amplitude of the coding fraction increases, especially at weak synaptic strengths, and the curve is almost flat for low and moderate synaptic values. Hence, very slow signals can be very well encoded by the network, even with little to no noise (notice the coding fraction axis has been extended to 1 in Fig. 5b). The reason is that the signal is so slow and its power is contained in such a small frequency range, that it can be easily sampled by the population of neurons with a firing rate of around *r*=0.3. For slow signals, an increase in the synaptic amplitude only decreases the coding fraction, i.e. in this case we do not observe SSR. Very slow signals, however, are probably less relevant for real neural systems.

## Feedforward control experiments

The previous sections showed that a recurrence-mediated improvement of signal encoding occurs across a large swath of the parameter space. We hypothesized that the underlying mechanism is SSR, only that here the recurrent input acts as network noise in place of the intrinsic white noise used for SSR in feedforward networks. In this interpretation, an increase in synaptic strength *J* will intensify the network noise, and thus *J* replaces the noise intensity parameter in conventional SSR curves. However, in addition to the network noise, changing the connection strength of neurons alters other statistics of the network, e.g. the firing rate of the neurons. In the following, we demonstrate through control simulations of an equivalent FFN, that it is indeed the increase in network noise that causes the improved signal transmission. The FFN is for all parameter values the same as the recurrent model in Eq. (1) except that $J=0$ and the bias $\mu$ and/or the intrinsic noise level $D_i$ are altered as discussed in the following.

**Fig. 6:**
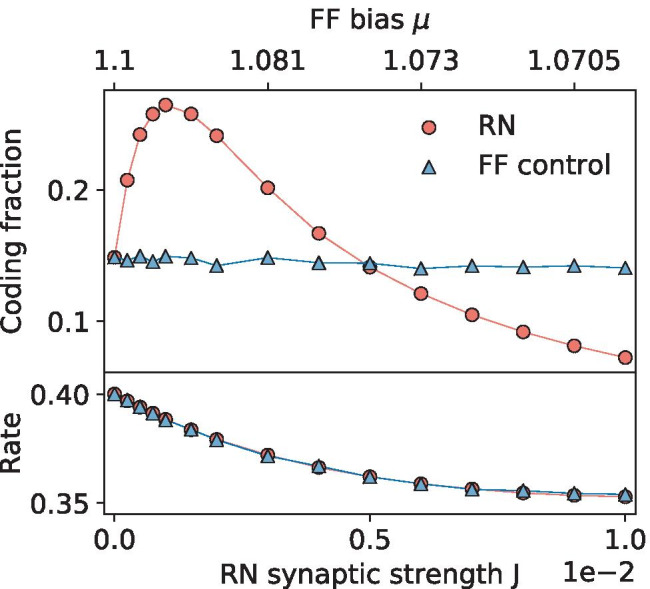
**Control for the effect of a changing mean from synaptic input.** The bias $\mu$ of a feedforward network (FF control) is adjusted to match its firing rates to those of a recurrent network (RN), as shown in the bottom panel. Using the new bias, the FFN’s coding fraction is compared to an equivalent RN’s (all parameters are equal except those that control synapses). **RN Parameters:** $\mu$=1.1, $D_i$=$2.5\times 10^{-5}$, *g*=5, $p_c$=0.01, $N_A$=250, $\sigma _s$=0.1, $f_c$=15

### Controlling for the firing rate

The simplest control is to adjust the firing rates of the neurons in the FFN through their bias term $\mu$. This would rule out a change in coding fraction due to the network sampling the stimulus at a higher rate. Figure 6 shows that, although the rates of the FFN are adjusted, the coding fraction is almost stagnate. In fact, as one would expect, as the bias decreases and the rate along with it, the coding fraction also decreases, but the change is so subtle that it is not immediately obvious from the blue curve in the top panel without zooming in. It should also be noted that the bias is changed only very slightly, resulting in less than a 10% decrease in the firing rate. However, this only gives further evidence to the claim that the firing rate of the RN is not responsible for the drastically better coding fraction performance.

### Controlling for the network noise with a diffusion approximation

Given that the firing rate is not responsible for the SSR, the next step is to take into account the ’network noise’ in a control experiment with an FFN. Roughly, the recurrent synaptic inputs can be approximated by a Gaussian white noise process using the so-called *diffusion approximation* (DA) following the approach in Brunel (2000). In contrast to the latter, however, for the control we do not use a self-consistent rate from the mean-field theory, but instead the measured average firing rate of the recurrent network, $r_\text {RN}$. For small synaptic weights, recurrent inputs in the asynchronous irregular state can be approximated by the sum of a time-independent mean

11 $$\begin{aligned} \mu _R&= J C_E r_\text {RN} \tau - g J C_I r_\text {RN} \tau \\ \nonumber&= J C_E (1 - \gamma g) r_\text {RN} \tau \end{aligned}$$

and a white noise term with intensity

12 $$\begin{aligned} D_R = \frac{1}{2} J^2 C_E (1 + \gamma g^2) r_\text {RN} \tau , \end{aligned}$$

giving a total mean input of $\mu ' = \mu + \mu _R$ and total noise intensity $D_i'$=$D_i+D_R$, the values which are used in the FFN simulations.

**Fig. 7:**
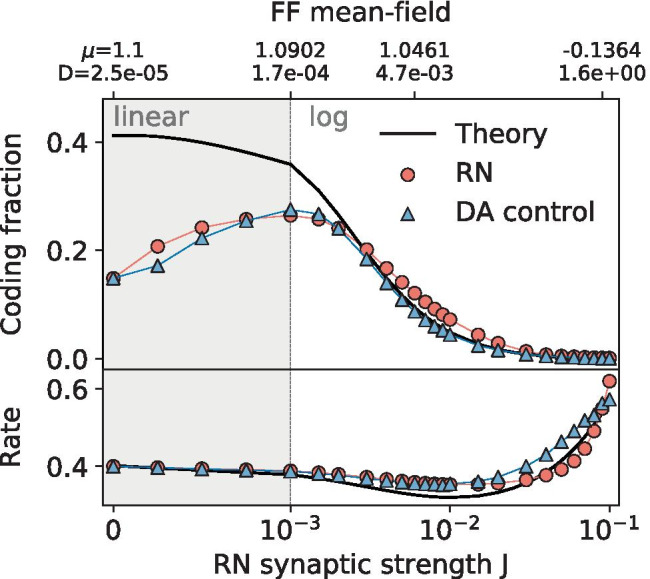
**Control for the effect of a changing mean and noise intensity from synaptic input.** The mean input and intrinsic noise intensity of the FFN are adjusted using the diffusion approximation of the recurrence (Eq. (11) and Eq. (12) with the measured RN average firing rate) in order to compare the effects of network noise and diffusive white noise on encoding. **Top:** The FFN’s coding fraction (DA control; blue triangles) is plotted with the RN’s (red points) and DA linear response theory (black line). **Bottom:** The measured firing rates of the networks (same symbols as above) compared to the self-consistent theory in Eq. (13) (black line). Parameters same as in Fig. 6

The FFN’s coding fraction (blue triangles and indicated as ’DA control’ in Fig. 7) very closely resembles that of the RN’s (red circles), demonstrating that the coding fraction benefit and SSR results seen thus far in the paper can be explained largely as the result of the extra noise provided by the network input.

Limitations of this approximation have different origins:

i) pronounced temporal correlations emerge self-consistently in many situations (Lerchner et al., 2006; Dummer et al., 2014; Vellmer & Lindner, 2019), even when synaptic filtering is neglected (which adds another low-pass filter, see e.g. Brunel & Sergi, 1998; Lindner & Longtin, 2006; Moreno-Bote & Parga, 2010), i.e. the network noise is colored and not white;
ii) synaptic input for larger amplitudes is poorly approximated by Gaussian noise (Richardson & Gerstner, 2005; Wolff & Lindner, 2008), which can have strong effects on the neural firing statistics (Richardson & Swarbrick, 2010; Droste & Lindner, 2017a). For the relatively low synaptic amplitudes considered here, the second limitation is not significant, but the first plays a role.

**Fig. 8:**
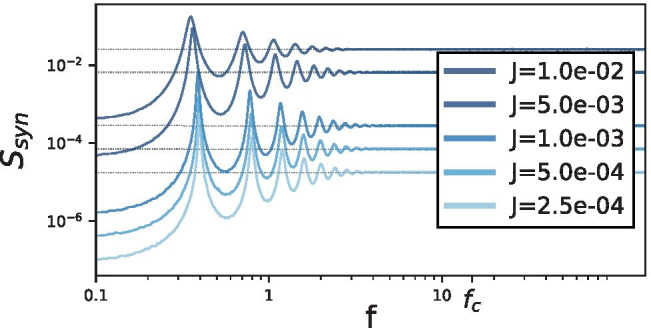
**Synaptic input power spectra.** The blue lines show the measured synaptic input power spectra.  The dashed lines are the mean-field white-noise approximations of the recurrent spectra, $S_\text {DA} = 2 D_R$, with the noise intensity from Eq. (12). Parameters same as in Fig. 6

In fact, in line with previous studies of correlations in network noise (Lerchner et al., 2006; Dummer et al., 2014; Wieland et al., 2015), we find that the power spectrum of the synaptic input (i.e. of the network noise) is far from being spectrally flat (see Fig. 8). For large frequencies, it is indeed flat because it is a sum of spike trains, the delta functions of which create a flat spectrum at high frequencies in the Fourier domain. At low and intermediate frequencies, however, we find decreased power or peaks, respectively. With an increase in the synaptic amplitude, the high-frequency limit increases and the peaks broaden, which is an effect of the network noise. Hence, small deviations in the coding fraction of the control feedforward system and of the RN as observed in Fig. 7 can be expected based on the difference in the statistical nature of the background fluctuations.

**Fig. 9:**
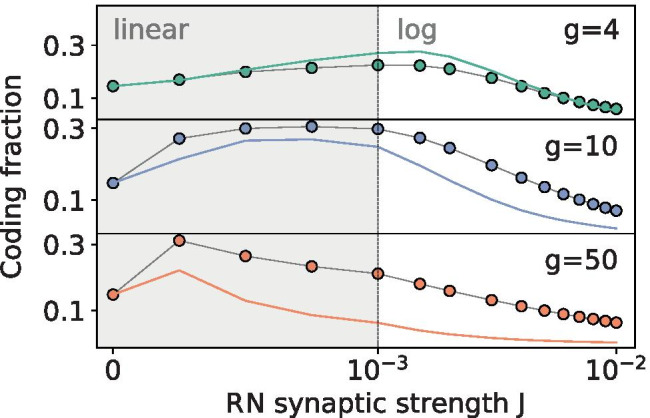
**Recurrence outperforms DA as inhibition dominates.** The feedforward DA control (colored, solid lines) superimposed over the RN data points (circles) for balanced (top: *g*=4) and inhibition-dominated regimes (middle: *g*=10; bottom: *g*=50). Other parameters same as in Fig. 6

As seen in Fig. 7, this difference between the actual synaptic input power spectrum and the DA has a somewhat negligible effect on the firing rate, except for very high synaptic weights (see lower panel), but does cause deviations between the curves on either side of the SSR peak (upper panel). In order to further explore these differences, the DA control was run for increasing values of *g*, as shown in Fig. 9. Interestingly, for a balanced net, the DA captures the coding fraction on either side of the peak quite well and worse at the peak itself, whereas for higher *g* values it does consistently worse. For *g*=50 (an extreme and unrealistic value from a physiological point of view), the DA control performs significantly worse, indicating that the recurrence is no longer well-approximated by white noise (here also the Gaussian approximation may be less justified), and more closely resembles a colored noise process. Remarkably, in almost all cases this colored noise difference from the DA is beneficial to the encoding, outperforming the white noise in all inhibition-dominated regimes. This demonstrates that the type of correlated noise generated by the recurrent network may be especially useful for signal processing.

Because the RN behaves similarly to the FFN with adjusted mean input and intrinsic noise level, it is worth asking if existing DA theories can be applied to predict the coding fraction of an RN. First of all, in the mean-field (MF) theory one can estimate the firing rate by a self-consistent solution of the classical equation for an LIF neuron receiving a mean input $\mu _\text {MF}$ and a Gaussian white noise with intensity $D_\text {MF}$ (Ricciardi, 1977), in which $\mu _\text {MF}=J C_E (1 - \gamma g) r_\text {MF} \tau$ and $D_\text {MF} = (1/2) J^2 C_E (1 + \gamma g^2) r_\text {MF} \tau$ depend on the firing rate (Brunel, 2000):

13 $$\begin{aligned} r_{\text {MF}} = \Bigg [ \tau _{\text {ref}} + \sqrt{\pi } \int _{\frac{\mu _\text {MF} - v_T}{\sqrt{2D_\text {MF}}}}^{\frac{\mu _\text {MF} - v_R}{\sqrt{2D_\text {MF}}}} e^{z^2} {{\,\mathrm{erfc}\,}}(z) dz \Bigg ]^{-1}. \end{aligned}$$

This yields a good description of the firing rate for neurons in the network, as seen by the black curve in the lower panel of Fig. 7.

Beyond the firing rate, the approach in Beiran et al. (2017) is applied to find linear approximations to the cross-spectrum, power spectrum, coherence and coding fraction of a population of feedforward LIF neurons with intrinsic Gaussian white noise. For the formulas we refer the interested reader to the exhaustive explanation by Beiran et al. (2017). As seen in Fig. 7, the DA analytical expressions are only able to describe the networks’ encoding for relatively large synaptic strengths (the axis is also extended to *J*=0.1 here), where the coding fraction is already declining. The reason for this is that for small weight values, there is not enough noise in the system to linearize it, and the theories are linear approximations; see the corresponding discussion by Beiran et al. (2017). Describing the coding fraction for a population of neurons with a low level of noise (no matter whether it is intrinsic or network noise) is an unsolved theoretical problem. For instance, at *J*=0 the network’s response to the signal is so nonlinear (Fig. 2a) that a perturbation theory is inappropriate to estimate it. As a consequence, it is unfortunately not possible to predict analytically where exactly the maximum of the coding fraction will be attained.

## Discussion

The question that was being investigated was whether the benefits to signal encoding, especially suprathreshold stochastic resonance (SSR), awarded by the increase in intrinsic white noise and the heterogeneity of parameters for feedforward networks (Stocks, 2000; Stocks & Mannella, 2001; Hoch et al., 2003; Das et al., 2009; Ashida & Kubo, 2010; Nikitin et al., 2010; Durrant et al., 2011; Hunsberger et al., 2014; Beiran et al., 2017) could also be found in recurrent networks whose synaptic weights were increased instead. SSR was shown to occur with respect to the level of network noise controlled by the synaptic coupling strength and the effect was robust over a wide range of intrinsic, network, and signal parameters.

Previous studies have shown that network input can be roughly approximated in some cases as white noise using the diffusion approximation (Gluss, 1967; Johannesma, 1968; Capocelli & Ricciardi, 1971; Ricciardi, 1977; Brunel, 2000). However, for all synaptic weights used, pronounced temporal correlations are present in the network (Ostojic, 2014; Wieland et al., 2015; Pena et al., 2018), as can be seen for our model in Fig. 8. We therefore tested to what extent these deviations of the network fluctuations from white noise influence the signal encoding of the recurrent network by comparing its coding fraction to that of an FFN receiving purely white noise. In general the network noise coding fraction was comparable to its DA feedforward counterpart, but where they differed the network noise mostly boosted performance for equivalent firing rates. In order to study the effect of colored network noise on the coding fraction, future studies could exploit extended mean-field theories involving Markovian embedding via a multidimensional Fokker-Planck equation (Vellmer & Lindner, 2019) or simplified rotator neurons (Van Meegen & Lindner, 2018).

In the recurrent network, intrinsic white noise was also used, albeit very little in order to provide a baseline stochasticity, and it was shown that increasing it improves the performance for only small synaptic values, where the network noise is not yet strong enough to overpower it. From previous work it is then assumed that a weak heterogeneity in thresholds would have a similar effect as such weak intrinsic noise. However, in large recurrent networks the main share of variability originates from the network noise, and thus we conclude that SSR due to network noise is more likely than SSR due to (comparatively weak) intrinsic noise.

The level of synaptic connection strength at which the SSR maximum was attained here is at the lower end of the physiological range. To see this, we have to convert our nondimensional voltage variables to physiological units by rescaling them by the reset-to-threshold difference, which is typically 10-30 mV. Were 30 mV to be used, for example, the scale for the synaptic coupling constant *J* would also be converted to units of evoked PSP amplitude, and hence the range of values of the synaptic amplitude that maximizes the coding fraction (in nondimensional units between $10^{-3}$ and $10^{-2}$) would correspond to $0.03-0.3$mV for excitatory synapses. Because in our homogeneous system, we use the same constant amplitude *J* for all excitatory connections, this must be compared to the average excitatory PSP. The estimates of this average PSP vary widely, depending on species and brain region. For instance, in young rat somatosensory cortex estimates are around 1.3 mV (Markram et al., 1997), whereas in the hippocampus of guinea pigs it is on the order of 0.1mV (Sayer et al., 1990). Despite our strongly simplified setup of a sparse, random and homogeneous connectivity, we may nonetheless naively conclude that the network fluctuations provide more than enough network noise to cover the breadth of biological estimates as required for the SSR effect, and may even be so high as to be detrimental to signal encoding.

This picture may change if we take into account heterogeneity of synaptic amplitudes (Lefort et al., 2009), correlations between activity and connectivity (Yassin et al., 2010), and other non-random features in network connectivity (Song et al., 2005). First of all, a heterogeneity of amplitudes would also provide a source of noise if they were drawn from a distribution, as quantified recently by us and others (Bostner et al., 2020). Going beyond a purely randomly drawn connectivity, for instance specific topologies (Litwin-Kumar & Doiron, 2012) or the overrepresentation of bidirectional connections (Esposito et al., 2014; Brunel, 2016), will also influence where the SSR maximum with respect to the synaptic amplitude is attained; this can be taken into account by mean-field methods, developed for instance in Refs. Schmeltzer et al. (2015); Laing and Blaesche (2020). Future studies may reveal the way in which such correlations act on the transmission of sensory stimuli.
